# Musical Training in the Development of Empathy and Prosocial Behaviors

**DOI:** 10.3389/fpsyg.2021.661769

**Published:** 2021-05-11

**Authors:** Xiao Wu, Xuejing Lu

**Affiliations:** ^1^CAS Key Laboratory of Mental Health, Institute of Psychology, Chinese Academy of Sciences, Beijing, China; ^2^Department of Psychology, University of Chinese Academy of Sciences, Beijing, China

**Keywords:** empathy, development, neuroimaging, music training, prosocial behavior

## Abstract

Music not only regulates mood but also promotes the development and maintenance of empathy and social understanding. Since empathy is crucial for well-being and indispensable in social life, it is necessary to develop strategies to improve empathy and prosocial behaviors. To fulfill this aim, researchers have extensively investigated the effect of intensive musical training on the development of empathy. Here, we first summarize evidence showing the powerful influence of musical training on the development of empathy and then discuss psychological mechanisms responsible for those observations. The conclusions drawn from most previous studies were mainly based on behavioral measurements, while the neural basis of musical training in the development of the empathic brain is still unclear. Fortunately, brain imaging research has contributed greatly to our understanding of the neural underpinnings associated with musical training and its possible connection to the development of the empathic brain. One of the most distinctive signatures of musical training is structural and functional changes of multiple brain regions, and such changes might be related to some of the empathic behaviors observed in musically trained children. Therefore, intensive musical training in childhood may increase levels of empathy, and applied research is required to optimize the training strategy before implementing music education in empathy regulation. Moreover, future longitudinal studies are needed to better understand neural mechanisms underlying the causal effect of musical training on empathy development. These findings have important implications for understanding the development of the empathic brain and for improving prosocial behaviors.

## Musical Training Boosts Empathy and Prosocial Behaviors

Empathy is a multidimensional construct that relates to prosocial behaviors and altruism among both adults and children (Eisenberg and Miller, 1987). Empathy has been considered as a strong predictor of prosocial behaviors (Roberts and Strayer, 1996). Empathy includes at least three related but distinct components – affective (feeling others’ emotions), cognitive (taking others’ perspective), and motivational (desiring to promote others’ well-being or alleviating their suffering) components (Decety, 2015; Weisz and Cikara, in press), based on shared and distinct neural systems (Kogler et al., 2020). Empathy is crucial for well-being and indispensable in social life. When an individual can experience other’s feelings and adopt other’s perspectives, he or she is likely to avoid causing any harm (van Hazebroek et al., 2017). Indeed, it has been suggested that the levels of empathy and the development of aggressive and violent behaviors are highly correlated – lower levels of empathy increase the development of manifest and relational aggressiveness, while high levels of empathy decrease the emergence of violent acts (van Hazebroek et al., 2017; Arufe-Giraldez et al., 2019). Using structural equation modeling analysis, a recent investigation on a cohort of 734 school children aged 10–12 years demonstrated a negative relationship between affective empathy and relational aggression. These findings suggested that the level of empathy is a protective factor against the development of violent behaviors in schoolchildren (Castro-Sanchez et al., 2019). Viewed from this standpoint, empathy is a desirable personal characteristic and might be an accelerator for prosocial behaviors; therefore, a deliberate regulation on empathy appears to be necessary, especially for those with empathy deficits, as it would lead to enduring changes to individuals’ emotional and social lives in the real world. Early theorists assumed that young children were self-centered and cognitively incapable of experiencing empathy (e.g., Piaget, 1965). This view is now being challenged, as accumulating evidence showing that even young infants can experience concern for others with the ability to differentiate between self and others (Davidov et al., 2013). Although empathy emerges and develops early in life, it is flexible and amenable to behavioral interventions (Decety, 2015). McDonald and Messinger (2011) identified both intra-individual contributors (e.g., genetics, neural development, and temperament) and socialization factors (e.g., facial mimicry and imitation, parenting, and parent-child relationships) that influence the development of empathy in a young child (see also Ornaghi et al., 2020).

As one of the behavioral interventions, music is capable of promoting the development and maintenance of empathy and social understanding through its powerful affective, cognitive, and social components (Clarke et al., 2015). When listening to music, people often connect what they have heard to their thoughts and emotions. This process is associated with the concept of “theory of mind” – the ability to understand the intentions and emotional state of others (Jackendoff and Lerdahl, 2006; Perlovsky, 2010). Indeed, empathy is putting oneself in other people’s shoes and feeling what one thinks others are feeling, but maintaining the distinction between self and others, while listening to music involves a similar process of viewing from a distance as in empathy (Epperson, 1967). In other words, a person usually experiences feelings while listening to music but remains an observer at a distance (Kalliopuska and Ruókonen, 1986). When we engage in music activities, we perceive the emotional and psychological content of music, and interpret the thoughts and feelings of others (Greenberg et al., 2015). Researchers have also suggested that music and empathy are closely linked (Eerola et al., 2016; Huron and Vuoskoski, 2020). For instance, both perceived and induced emotion from music can be moderated by empathy (Egermann and McAdams, 2013), and empathy can also modulate physiological reactions to music (Miu and Balteş, 2012). In addition, numerous empirical studies have shown that long-term musical training has a positive influence on children’s empathy, sympathy, and prosocial behaviors (Schellenberg et al., 2015). For instance, Kirschner and Tomasello (2010) examined the relationship between collaborative music activity and helping behaviors in 4-year-old children, and they found that children who synchronized with peers showed more helping behaviors than children who only chatted with peers. Similarly, after a 3-month musical education program (1 h/week), the holistic empathy of children aged 6–7 years significantly improved (Kalliopuska and Ruókonen, 1986, 1993). Comparing students aged 10 years in average (age range: 8–11 years) with and without music-group interactions for an entire school year (i.e., performing various musical tasks in the form of pre-arranged musical games, e.g., entrainment games that encourage students to be as rhythmically coordinated with others as possible vs. participating in similar games but with no use of music, e.g., verbal story-telling, drama, and other forms of interaction), Rabinowitch et al. (2012) found that children with music-group interactions showed higher emotional empathy scores than control children at the end of the study.

In addition to empathy *per se*, adults with professional musical training have heightened sensitivity to emotions in speech compared to non-musicians (Kraus and Chandrasekaran, 2010), and such increased sensitivity associated with musical training can be extended to non-speech vocalizations, e.g., infant crying (Parsons et al., 2014). Also, active music activities are effective in improving the executive function of children aged 3–4 years (Bowmer et al., 2018) and social competence in children and adolescents with social deficits (Gooding, 2011; Greenberg et al., 2015). Given that a range of studies reporting strong correlations between executive function/social competence and empathy development (Sallquist et al., 2009; McDonald and Messinger, 2011; Yan et al., 2020), it also suggests an essential role of musical training in the development of empathy. More importantly, it seems that the starting age of musical training is critical in the development of empathy. An earlier onset of musical training (i.e., aged ≤7 years) not only yielded greater cognitive and motor abilities (Watanabe et al., 2007; Bailey and Penhune, 2013) but also boosted the development of empathy. For example, Kawase et al. (2018) conducted a survey of 276 children aged 4–5 years and 6–7 years who started musical training at 1, 2, 4, and 6 years old. The results showed that the empathy scores of children aged 6–7 years who began training at 1 year old were greater than those who started at 4 years old, suggesting that early onset of music training positively influenced children’s empathy. All these observations introduce an exciting possibility – the development of empathy can be facilitated by musical training in childhood, thus promoting prosocial and altruistic behaviors.

## Psychological Mechanisms of the Effect of Musical Training in the Development of Empathy

A core issue following the aforementioned studies is to clarify the underlying mechanisms of the effect of musical training in the development of empathy. Empathy is built through processes similar to those involved in music playing, including sharing affective experiences, imitation, being synchronous, and collaborating (Rabinowitch et al., 2012). At present, a great number of studies have uncovered distinct emotional and cognitive mechanisms underlying the musical training effect in the development of empathy. During music practice, children develop affective and cognitive abilities necessary for empathic competence functions. For instance, musically trained children tend to be more sensitive to emotions expressed in music (Castro and Lima, 2014) and more likely to share the affective experiences of others’ actual or inferred emotional state (Kalliopuska and Tiitinen, 1991). This observation can be attributed to the powerfully emotional benefits associated with musical training. Since music is inevitably linked with feeling, expressing, and perceiving emotions (Juslin and Vastfjall, 2008), it makes perfect sense to hypothesize that musical training could be predictive of improved emotional abilities in childhood. Indeed, children are surrounded by a world of rich emotional experiences when playing music, and such experiences provide the basis for emotion recognition and experience sharing.

On the other hand, the positive association between musical training and emotional abilities might be a consequence of high levels of cognitive functioning among the musically trained children (Schellenberg and Peretz, 2008). Take learning to play a musical instrument, for example. It is a demanding task that develops sophisticated sensory and motor skills (Zatorre et al., 2007), which provides a neurocognitive basis for physical mimicry of others’ movements and emotions (Cross et al., 2012). Empathy is built upon the experience of one’s own body and associated with the mirror neuron system (Rizzolatti et al., 2001). The mirror neuron system is sensitive to both action observation and movement learning, which is established through the operation of associative processes (Heyes, 2010) and highly related to the sensorimotor process (Giacosa et al., 2016). Perception of a given behavior initiated by another individual automatically activates one’s own representations of that behavior and further helps one form similar feelings and thoughts of others (Knoblich and Flach, 2003). Music activities typically involve a massive sensorimotor process related to action execution and observation, which are important for empathy (Li et al., 2019). Therefore, with the enhanced association between the perception and execution of actions, children with intensive musical training are more likely to represent and imitate others’ movements. Such action representation could further modulate emotional activity, which may provide an essential functional architecture for empathy (Carr et al., 2003).

More importantly, playing music promotes contact with others and acts as a vehicle for social interaction. In fact, social interaction is also associated with the mirror neuron system (Clarke et al., 2015), given that the mirror neuron system is built through sensorimotor experiences (Heyes, 2010), and most experiences are obtained through interaction with others. Compared with passive music activity (i.e., music listening), active music lessons for 6-month-old infants facilitated communications and social interactions with parents (Gerry et al., 2012). Music activities also promote synchrony. Synchronization among group members is suggested to be a key to promoting sociability (Weinstein et al., 2016; Kawase et al., 2018). Both social interaction and synchrony contribute to cultural identity and encourage the formation of cooperative networks. Since empathy is shaped by social context (Decety, 2015), it appears that music can act as an agent of social bonding and affiliation (Huron, 2001) and boost empathic behaviors accordingly (Clarke et al., 2015).

## Brain Structural and Functional Changes Associated with Musical Training Support the Development of the Empathic Brain

The possible psychological mechanisms discussed above were mainly based on the evidence from behavioral studies, which cannot reveal the neural basis of the relationship between musical training and empathy development. Fortunately, brain imaging research has contributed greatly to our understanding of the neural underpinnings associated with musical training. From an evolutionary perspective, music likely “piggybacked” upon the neural systems that evolved to help us share others’ feelings and navigate our social world (Wallmark et al., 2018). Although the brain structural and functional changes associated with musical training do not necessarily guarantee a significant impact on empathy, it is suggested a possible connection between musical training and the development of the empathic brain.

Over the last few decades, a considerable body of neuroimaging studies have demonstrated that one of the most distinctive signatures of musical training is structural changes in brain regions, such as auditory (Gaser and Schlaug, 2003; Zatorre, 2005; Bangert and Schlaug, 2006; Elmer et al., 2013), and sensorimotor areas (Gaser and Schlaug, 2003; Jancke, 2009), as well as regions not primarily engaged in music activities, e.g., inferior frontal regions (Peretz, 2002), multimodal integration regions (Bangert and Schlaug, 2006; Zatorre et al., 2007), and the corpus callosum (Schlaug et al., 1995; Schmithorst and Wilke, 2002; Bengtsson et al., 2005; Hyde et al., 2009). These structural differences seem to be more evident in musicians who began training at an early age (Schlaug et al., 1995; Hyde et al., 2009) and in musicians who practice more intensively (Gaser and Schlaug, 2003). These structural changes associated with intensive musical training starting in early life are paralleled by improvements in auditory processing, sensory-motor skills, and higher-order cognitive functions (Schlaug et al., 2005; Fujioka et al., 2006; Zatorre et al., 2007; Hyde et al., 2009; Seither-Preisler et al., 2014; Habibi et al., 2018; see Penhune, 2011 for a review), and we infer that they could be important neural bases prepared for the development of empathy.

To examine associations between developmental changes in the brain and musical training, Hudziak et al. (2014) used longitudinal data from the large NIH-MRI study of Normal Brain Development and showed that healthy developing children playing a musical instrument exhibited more rapid changes in cortical thickness within areas associated with motor planning and coordination when compared with children not playing a musical instrument. However, one may argue that the group differences of cortical thickness changes are mainly attributed to the pre-existing differences between the two groups since no pre-training data were acquired in this study. This possibility can be ruled out indirectly by another study, in which Schlaug et al. (2005) reported no pre-existing brain structural differences in a group of 6–7 years old children who were about to begin musical training and their matched counterparts who were not intending to take music lessons. After 15 months of musical training, children in the instrumental group showed structural changes, including increased gray matter density in the primary auditory and motor areas, and increased volume of the corpus callosum, while these changes were not seen in the children who did not receive musical training (Hyde et al., 2009). Another line of evidence was from a two-stage study conducted by Habibi et al. (2014). First, Habibi et al. (2014) observed no pre-existing differences among three groups of children aged 6–7 years in terms of cognitive, motor, musical, emotional, and social behavior measures as well as in structural and functional brain measures. Two years later, when compared with those in the sports group and no training group, they found that the rate of cortical thickness maturation of the right posterior superior temporal gyrus differs from that of the left side among children in the music group. Moreover, musically trained children exhibited higher fractional anisotropy in the corpus callosum, specifically in the crossing pathways connecting superior frontal, sensory, and motor segments (Habibi et al., 2018). In other words, musical training induces brain changes at both macro (e.g., volume, thickness, and surface area of gray matter) and microstructural (e.g., fractional anisotropy in the corpus callosum) level in school-age children, and these changes cannot be attributable to pre-existing biological differences.

In addition to structural alterations associated with intensive musical training, a large body of studies have demonstrated that musical training alters the functional connectivity (FC) between brain regions that are typically involved in music perception and production, including sensory, motor, multisensory, and cognitive regions of the cortex (Fauvel et al., 2014), as well as subcortical brain regions (Schmithorst and Wilke, 2002; Zamorano et al., 2017; Li et al., 2019; Zamorano et al., 2019), and even the cerebellum (Schmithorst and Wilke, 2002). Musicians showed either hyperconnectivity (Fauvel et al., 2014; Klein et al., 2016; Zamorano et al., 2017) or hypoconnectivity (Imfeld et al., 2009), depending on the brain regions or tracts (Schmithorst and Wilke, 2002; Bengtsson et al., 2005). For instance, Li et al. (2019) found increased insular FC in musicians. More importantly, there were significant associations between discrepant insular FC patterns and empathy scores in musically trained people, suggesting an enhancement of musical training on insular subnetwork functions, e.g., empathy. It has been suggested that the insula plays a vital role in processing subjective feelings and emotions (Craig, 2009). Furthermore, the activation of the insula is related to the process of linking observed expressions of emotions with internal empathic responses (Carr et al., 2003), and the degree of activation during emotion processing tasks is shown to be positively correlated with measures of empathy (Singer et al., 2004). Since empathy relies on the ability to internally simulate perceived emotions (Lamm et al., 2019), the observed enhancement of musical training on insular subnetwork function suggests that musical training would facilitate the integration of interoceptive and exteroceptive information and result in better affective sensitivity and empathic ability (Li et al., 2019).

## Implications and Future Directions

The brain imaging studies we summarized above showing that musical training in childhood has profound and constructive effects on the structure and function of the brain. As described, intensive musical training can lead to modifications of brain structure and function likely associated with the development of empathy. These findings have important implications for understanding the development of the empathic brain and for improving prosocial behaviors. First, it is increasingly appreciated that music activities are useful therapeutic tools for children with empathy deficits, e.g., children with autism spectrum conditions (ASC, Greenberg et al., 2015). Individuals with ASC often have difficulties in emotion perception, expression, and communication. Music, as a potential medium that encourages ASC individuals to engage in social interactions, may facilitate social connection through a wide range of music activities. For instances, after a 52-week active music therapy (60 min/week) that consists of a wide range of music activities, including singing, piano playing, and drumming, the severity of psychiatric conditions of young adults with severe autism (e.g., lack of interaction with peers or therapists) rated by psychiatrists was significantly decreased (Boso et al., 2007). Since musical training is an engaging and enjoyable activity, it is easy to implement in special groups, and its benefits outweigh its inputs. Second, given that group music lessons include multiple interactions among peers and parents, they require various types of social interactions than one-to-one lessons. Such social interactions in group music lessons may provide a comprehensive context to facilitate children’s sociability. Therefore, group music education could be considered as one of the main approaches for the development of empathy and prosocial behaviors. Last, the ultimate goal of emotional education associated with empathy is to teach children how to perceive the emotions of their own and those of other people sensitively, and accordingly to develop different aspects of empathy and a variety of strategies to face different emotional situations. From this perspective, music activities for emotional education have clear advantages, as, for example, music can be experienced as sad without becoming sad oneself. In other words, music can increase levels of empathy but not harm oneself.

In summary, musical training has long been viewed as an approach to regulate emotion and empathy – musical training is positively associated with the increased level of empathy and prosocial behaviors and negatively associated with aggressive acts and behavioral problems (e.g., Greenberg et al., 2015; Schellenberg et al., 2015; Li et al., 2019). However, several issues should be considered and addressed in future investigations. First, the efficacy of musical training on the development of empathy should be assessed quantitatively and objectively. To establish a causal link between musical training and empathy improvement, longitudinal studies with a strong neuroscientific basis are of great importance. Although cross-sectional neuroimaging studies provide information about the potential benefits of musical training on empathy, longitudinal studies allow stronger inferences to be made within a group of individuals since the possible influences of pre-existing individual factors (e.g., socioeconomic status) can be minimized. Second, in addition to musical training, other behavioral interventions are also reported to be effective in regulating empathy, e.g., meditation practices (Ashar et al., 2016). Therefore, from a practical point of view, future studies need to compare and combine different interventions to achieve the optimal outcome of empathy development. Third, empathy is a multicomponent construct, and different situations might require different components (Decety, 2015; Weisz and Cikara, in press). It is possible that various music activities (e.g., singing, instrument playing, and group training) are applicable to different populations, and they may contribute unequally to a specific component of empathy. Thus, applied research is required before implementing music education in empathy regulation extensively.

## Author Contributions

XW and XL conceived the idea and wrote the paper. Both the authors contributed to the article and approved the submitted version.

### Conflict of Interest

The authors declare that the research was conducted in the absence of any commercial or financial relationships that could be construed as a potential conflict of interest.
